# Traditional uses, phytochemistry, pharmacology and toxicology of garlic (*Allium sativum*), a storehouse of diverse phytochemicals: A review of research from the last decade focusing on health and nutritional implications

**DOI:** 10.3389/fnut.2022.929554

**Published:** 2022-10-28

**Authors:** Champa Keeya Tudu, Tusheema Dutta, Mimosa Ghorai, Protha Biswas, Dipu Samanta, Patrik Oleksak, Niraj Kumar Jha, Manoj Kumar, Radha, Jarosław Proćków, José M. Pérez de la Lastra, Abhijit Dey

**Affiliations:** ^1^Department of Life Sciences, Presidency University, Kolkata, India; ^2^Department of Botany, Dr. Kanailal Bhattacharyya College, Howrah, India; ^3^Department of Chemistry, Faculty of Science, University of Hradec Kralove, Hradec Kralove, Czechia; ^4^Department of Biotechnology, School of Engineering and Technology, Sharda University, Greater Noida, Uttar Pradesh, India; ^5^Department of Biotechnology, School of Applied and Life Sciences, Uttaranchal University, Dehradun, India; ^6^Department of Biotechnology Engineering and Food Technology, Chandigarh University, Mohali, India; ^7^Chemical and Biochemical Processing Division, ICAR-Central Institute for Research on Cotton Technology, Mumbai, India; ^8^School of Biological and Environmental Sciences, Shoolini University of Biotechnology and Management Sciences, Solan, India; ^9^Department of Plant Biology, Institute of Environmental Biology, Wrocław University of Environmental and Life Sciences, Kożuchowska, Poland; ^10^Biotechnology of Macromolecules, Instituto de Productos Naturales y Agrobiología, IPNA (CSIC). Avda, Astrofísico Francisco Sánchez, San Cristóbal de la Laguna, Spain

**Keywords:** allium sativum, traditional uses, ethnobotany, phytochemistry, pharmacology, toxicology

## Abstract

*Allium sativum* L. (Garlic) is a fragrant herb and tuber-derived spice that is one of the most sought-after botanicals, used as a culinary and ethnomedicine for a variety of diseases around the world. An array of pharmacological attributes such as antioxidant, hypoglycemic, anti-inflammatory, antihyperlipidemic, anticancer, antimicrobial, and hepatoprotective activities of this species have been established by previous studies. *A. sativum* houses many sulfur-containing phytochemical compounds such as allicin, diallyl disulfide (DADS), vinyldithiins, ajoenes (*E*-ajoene, *Z*-ajoene), diallyl trisulfide (DATS), micronutrient selenium (Se) etc. Organosulfur compounds are correlated with modulations in its antioxidant properties. The garlic compounds have also been recorded as promising immune-boosters or act as potent immunostimulants. *A. sativum* helps to treat cardiovascular ailments, neoplastic growth, rheumatism, diabetes, intestinal worms, flatulence, colic, dysentery, liver diseases, facial paralysis, tuberculosis, bronchitis, high blood pressure, and several other diseases. The present review aims to comprehensively enumerate the ethnobotanical and pharmacological aspects of *A. sativum* with notes on its phytochemistry, ethnopharmacology, toxicological aspects, and clinical studies from the retrieved literature from the last decade with notes on recent breakthroughs and bottlenecks. Future directions related to garlic research is also discussed.

## Graphical Abstract

Some of the pharmacological attributes of Garlic (*Allium sativum*). 
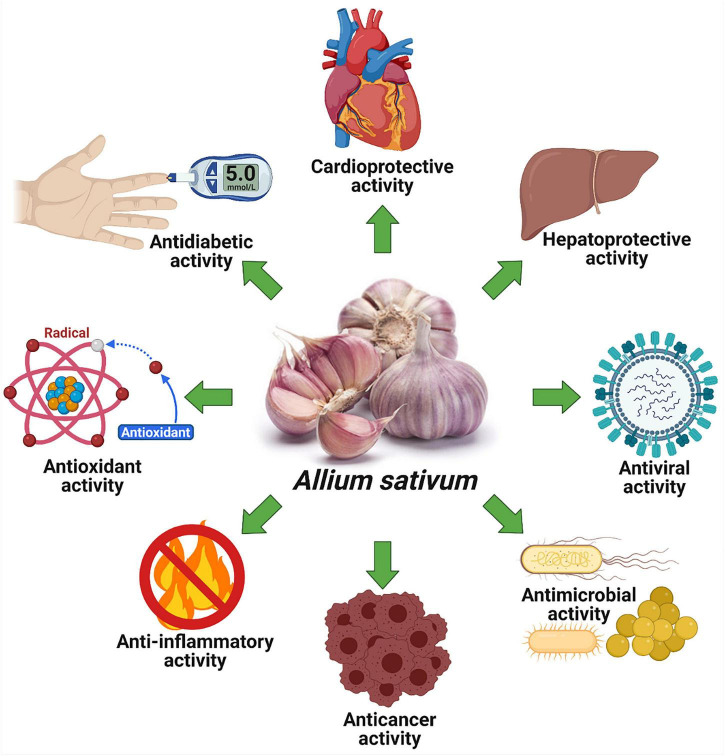

## Highlights

- Due to the numerous health benefits of *Allium sativum*, there is a growing interest in its use in various industries.
- Phytochemistry, ethnobotanical, and various pharmacological activities of *A. sativum* are extensively reviewed.
- *Allium sativum* contains various phytochemical compounds such as allicin, E-ajoene, and Z-ajoene that are of various therapeutic importance.
- Some of the sulfur-containing compounds extracted from *A. sativum* are reviewed along with their structures.
- Toxicological and clinical studies of *A. sativum* are summarized.

## Introduction

*Allium sativum* L. (Garlic) belongs to the family Amaryllidaceae, has been originated in Asia, and is also widely cultivated in Egypt, Mexico, China, and Europe (1). This plant is highly consumed in Iran, where its foliage, flowers, and cloves are employed in local medicine (1). All parts of *A. sativum*, bulbs, leaves, cloves, and flowers are utilized to prepare mixtures and decoctions to deal with various ailments. It is also a common spice and food additive. Studies on its phytochemistry indicate that sulfur-containing compounds, such as allicin, are the essential components. Allicin (diallyl-dithiosulfinate) is the most important alkaloid responsible for its beneficial effects. Other sulfur-containing phytochemicals found in *A. sativum* include diallyl disulphide (DDS), diallyl trisulfide (DTS), and S-allyl cysteine (SAC), which have a variety of pharmacological properties (1). In India, *A. sativum* is used to treat fever, coughs and is administered topically against scabies, graying of hair, and eczema, as well as against inflammation of the tetanus and lungs (2). In Pakistan, the plant extract is consumed orally against stomach ailments, respiratory problems, and fever. In Nepal, the Middle East and East Asia, the plant is applied against fevers, rheumatism, liver disorders, diabetes, colic, intestinal worms, dysentery, flatulence, tuberculosis, high blood pressure, facial paralysis, and bronchitis. In Africa, the plant has been reported to be an antibiotic, antiviral, hypolipidemic, hypoglycemic, and antithrombotic (2–4). In this review, the phytotherapeutic properties of garlic have been comprehensively investigated to provide an updated overall view of one of the most used (and best-selling) medicinal and food plants in the world with notes on ethnobotanical information validated by preclinical bioactivities (i.e., *in vitro* and *in vivo*), emphasizing the mechanisms and signaling pathways involved. We have also discussed the recent breakthroughs and bottlenecks of relevant research on garlic with notes on future perspectives on garlic research.

## Botany and geographical distribution of *Allium sativum*

**FIGURE 1:**
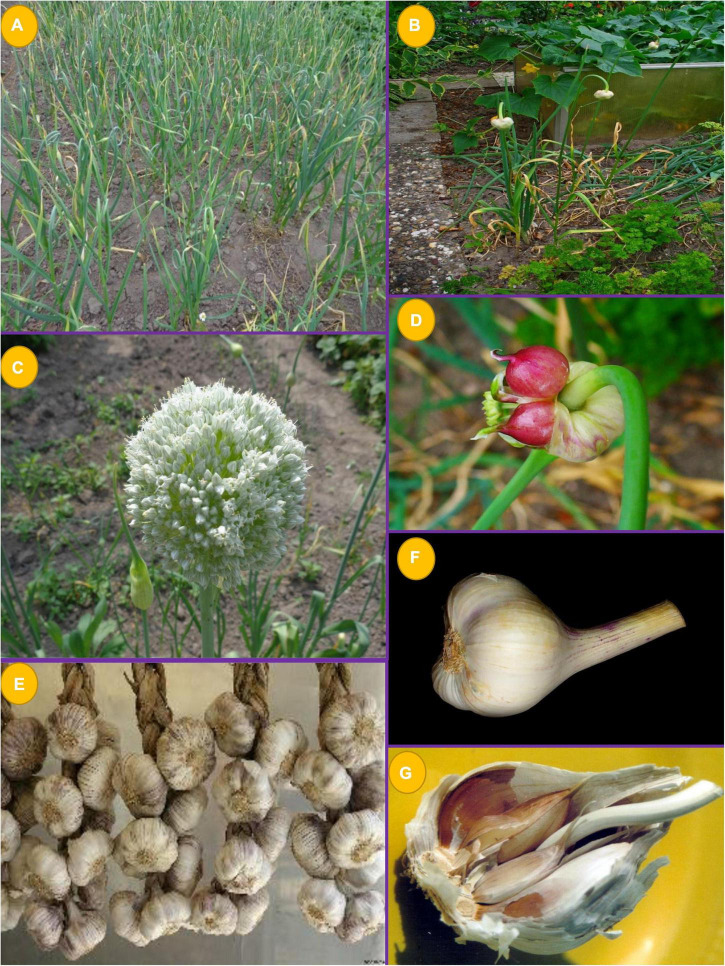
Different parts of garlic **(A,B)** habit; **(C,D)** flowers; **(E–G)** bulb (obtained from Wikimedia commons).

*A. sativum* L. (Figure 1), commonly known as garlic, belongs to the family Amaryllidaceae (5). The bulb is mostly used to treat ailments and the perennial herbaceous plant is large, with upright flowering stems that extend up to 1 m (6). The leaf blades are linear, flattened, robust, and approximately 0.5--1.0 inch (1.25--2.5 cm) long, with a pointed apex and violet to fuchsia flowers that bloom in the Northern Hemisphere during monsoons. Slender leaves on the exterior of the odoriferous bulb surround an internal sheath containing the cloves, and each bulb contains 10--20 cloves.^1^ Its medical benefits have been documented in Sanskrit texts dating back about 5,000 years and it first appeared in traditional Chinese medicine (TCM) at least 3,000 years ago (5). Today, garlic is grown almost everywhere and is known to have more than 300 varieties (7). At present, *A. sativum* is cultivated around the world. It was first discovered in Central Asia, then spread throughout China, the Near East, and the Mediterranean before making its way to the southern and middle parts of Europe, Mexico, and northern Africa, especially Egypt (5). Garlic is a perennial herb that thrives in mild regions and can be grown all year. Sowing each clove in the ground is a method to propagate the plant in cultivation asexually. Cloves are usually sown 6 weeks before the land freezes in the cold season. The bulbs only produce roots and have no stems above the surface.

## Phytochemistry of *Allium sativum*

**FIGURE 2:**
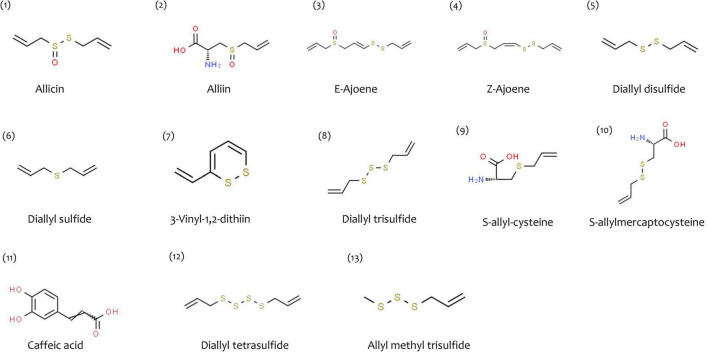
Structures of some of the phytochemicals reported from *A. sativum* (obtained from ChemSpider and PubChem).

*A. sativum* bulbs are reported to have many bioactive compounds, many of which are sulfur-containing, *viz.* thiosulfinates (allicin), sulfides [diallyl disulfide (DADS)], vinyldithiins (2-vinyl-(4H)-1,3-dithiin, 3-vinyl-(4H)-1,2-dithiin), ajoenes (*E*-ajoene and *Z*-ajoene) diallyl trisulfide (DATS), and so on constituting up to 82% of the total sulfur content in garlic. Allicin, S-methyl cysteine sulfoxide (MCSO), and S-propylcysteine sulfoxide (PCSO) are the main noxious compounds, with allicin being the predominant cysteine sulfoxide. Allicin, MCSO and PCSO, upon acted on by various enzymes, further produce molecules that include allyl methane thiosulfinates, methyl methanethiosulfonate and other thiosulfinates (8–10). Table 1 depicts a list of phytochemicals present in *A. sativum* with their molecular formula and IUPAC names. Figure 2 represents the chemical structures of phytochemicals (obtained from ChemSpider and PubChem).

**TABLE 1:** Phytochemistry of *A. sativum.*

Compounds	Molecular formula	IUPAC name	References
Allicin	C_6_H_10_OS_2_	3-prop-2-enylsulfinylsulfanylprop-1-ene	(7, 10, 65–67)
Alliin	C_6_H_11_NO_3_S	(2*R*)-2-amino-3-prop-2-enylsulfinylpropanoic acid	(7, 10, 65, 67, 68)
E-ajoene	C_9_H_14_OS_3_	(1E)-1-(Allyldisulfanyl)-3-(allylsulfinyl)-1-propene	(7, 10, 69)
Z-ajoene	C_9_H_14_OS_3_	(1Z)-1-(Allyldisulfanyl)-3-(allylsulfinyl)-1-propene	(7, 10, 65)
Diallyl disulfide (DADS)	C_6_H_10_S_2_	3-(prop-2-enyldisulfanyl) prop-1-ene	(7, 10, 68, 70, 71)
Diallyl sulfide (DAS)	C_6_H_10_S	3-prop-2-enylsulfanylprop-1-ene	(7, 10, 67, 68)
3-Vinyl-1,2-dithiin	C_6_H_6_S_2_	3-Vinyl-1,2-dithiine	(5, 7, 65)
Diallyl trisulfide (DATS)	C_6_H_10_S_3_	3-(prop-2-enyltrisulfanyl) prop-1-ene	(7, 10, 67, 68, 71)
S-allyl-cysteine (SAC)	C_6_H_11_NO_2_S	(2R)-2-amino-3-prop-2-enylsulfanylpropanoic acid	(5, 67, 68, 72, 73)
S-allylmercaptocysteine (SAMC)	C_6_H_11_NO_2_S_2_	(2R)-2-amino-3-(prop-2-enyldisulfanyl) propanoic acid	(67, 68)
Caffeic acid	C_9_H_8_O_4_	(2E)-3-(3,4-Dihydroxyphenyl) prop-2-enoic acid	(66)
Diallyl tetrasulfide	C_6_H_10_S_4_	3-(prop-2-enyltetrasulfanyl) prop-1-ene	(31, 70)
Allyl methyl trisulfide	C_4_H_8_S_3_	3-(methyltrisulfanyl) prop-1-ene	(70, 71)

## Ethnobotanical uses of *Allium sativum*

Garlic is a well-known culinary ingredient and seasoning due to its strong aroma, which is attributed to organosulfur compounds such as allicin and DADS. Garlic’s promising therapeutic advantages in ethnomedicine include its application against hypertension, pneumonia, hair loss, snakebite, diabetes, wounds, cough, paralysis, scabies, malaria, hemorrhoids, carbuncles, heart diseases, asthma, pain, respiratory disorders, influenza, female infertility, etc., which are mainly attributed to its antidiabetic, anti-atherosclerotic, antimicrobial, antihypertensive, anticancer, cardioprotective, diuretic, aphrodisiac, sedative, carminative, and antipyretic properties evidenced by various studies. Supplementary Table 1 shows the ethnobotanical and ethnotherapeutic uses of *A. sativum* with information on the place of the report, local names, using ethnic groups, modes of administration, preparatory techniques and applied dosages.

## Pharmacology of *Allium sativum*

The ethno-medicinal applications of *A. sativum* have been studied in a number of pharmaceutical studies and clinical investigations. The next section will gather the results of *in vitro*, *in vivo*, and *ex vivo* therapeutic studies using the plant to investigate pharmacological functions. The pharmacokinetic characteristics of *A. sativum* are shown in Table 2.

**TABLE 2:** Pharmacological investigations of *A. sativum*.

Pharmacological activity	Extract/fractions/plant parts	*In vitro*/*in vivo*/*ex vivo* assays/models	Underlying mechanism	References
Antioxidant activity	Raw garlic samples	DPPH radical scavenging assay, ABTS radical scavenging assay, FRAP assay	Inhibitory activity of the pro-oxidant enzyme, the ability to break radical chain propagation reactions, and β-carotene bleaching	(11)
	Ethanolic extract	DPPH assay, oxygen radical absorption capacity (ORAC) assays, HT22 mouse hippocampal neuronal cell line	Prevent Aβ- or glutamate-induced cell death, early stage of sprouting	(12)
	Ethanolic extract, distilled water extract	DPPH, ABTS, FRAP, H_2_O_2_ scavenging, and Fe^2+^ chelating assays	Flavonoids and phenolic compounds, fenton reaction	(13)
	Ethanolic clove extract	DPPH and superoxide radical assay	Scavenge H_2_O_2_, inhibition of lipid peroxidation	(14)
	Bulb extract of aged garlic	Human endothelial cells	Inducing the expression of several antioxidant enzymes, the ho-1 and GCLM subunit, through nrf2- are the pathway	(143)
	Saponin based methanolic extract	Mouse-derived c2c12 myoblasts	Scavenging intracellular reactive oxygen species	(144)
Hepatoprotective activity	Ethanolic clove extract	CCl_4_ induced male rabbits	Orally received 3 ml/kg of CCl_4_ in olive oil (1: 1) as 1/4 LD50, serum ALT, AST, ALP, TB, and TSP were determined	(14)
	Lactic acid-fermented garlic extract	Acetaminophen (AAP)-induced acute liver injury in rats	Suppressing MAPK phosphorylation, downregulating p53	(15)
	Lactic acid-fermented garlic extract	Alcohol-induced fatty liver damage in C57BL/6J mice	Decreased TBIL and DBIL values, low serum enzymes such as ALT, AST, and ALP	(145)
	Aqueous bulb extract	Wistar rats	Improving plasma biochemical factors of liver function, such as urea, creatinine, and aspartate transaminase and alanine transaminase	(17)
Anti-inflammatory activity	Aged garlic extract	Apolipoprotein E-knockout mice	Reducing the level of TNF-α and interleukin IL-1 receptor-associated kinase 4, increasing AMPK activity in the liver	(20)
	Hexane clove extract	Lipopolysaccharide-induced macrophage cell line RAW264.7	Down-regulating the expression of iNOS and COX2	(21)
	Bulb extract or Allicin	BALB/c mice with schistosomiasis (*S. mansoni* infection)	Expression of IL13, tTG, IL-1β, IL-6, and TNF-α, immunohistochemical expression of fibronectin, and α-SMA, mRNA expression	(22)
	Aqueous bulb extract	LPS induced J774A.1 macrophages	Inhibition of NF-kB transcription factor signaling pathway	(19)
Cardioprotective activity	Aged garlic extract	Isolated rat aortic rings	Stimulation of nitric oxide production, leading to endothelial-dependent vasodilation	(23)
	Fermented garlic extract by *Bacillus subtilis*	Spontaneous hypertension rats	Modulation of the sGC-cGMP-PKG pathway	(146)
	Fermented garlic extract	Monocrotaline-induced pulmonary hypertension rats	Decreasing the expression of vascular endothelial cell adhesion molecule-1 and MMP-9, increasing the expression of PKG and eNOS	(25)
	Black garlic extract (1.5%)	High-fat diet-fed male Sprague–Dawley rats	Reducing the mRNA expression of sterol regulatory element-binding protein-1c	(147)
	Aqueous garlic homogenate	Male Sprague–Dawley rats	Increasing Na^+^/K^+^-ATPase protein level	(148)
	Raw garlic	Streptozotocin-induced diabetic rats	Deacetylation of manganese superoxide dismutase, SIRT3 modulation	(149)
	Garlic extract powder capsule	Insulin-resistant obese rats	Cardiac mitochondrial ROS production, cardiac mitochondrial swelling	(150)
	Garlic extract	Rat model of gentamicin-induced chronic renal failure	Reducing oxidative stress, controlling Na^+^/K^+^-ATPase activity and Ca_2_ + levels	(151)
Anticancer activity	Ethanolic clove extract	DLD-1 human colon cancer cells	Down-regulating the expression of cyclin B1 and CDK1, inhibiting of activation of NF-kB	(26)
	Clove extract	Human prostate cancer (PC-3), colon cancer (Caco-2), breast cancer (MCF-7), liver cancer (Hep-G2), mouse macrophage cell line (TIB-71)	Inhibiting cell proliferation, inducing cell cycle arrest inducing apoptosis	(27)
	Aged garlic extract	Human gastric carcinoma cell line (SGC-7901)	Accumulating Bax, p53, and cytochrome C and decreasing the expression of Bcl-2, MAPK pathway	(28)
	Crude garlic extract	Human breast cancer cells (MDA-MB-231), human esophageal cancer cells (WHCO1)	Targeting the folding of proteins in the endoplasmic the reticulum of cancer cells	(152)
	Ethanolic bulb extract	Human colorectal carcinoma cell line (SW620)	Regulate the JNK and p38 MAPK pathways, reducing cell viability	(153)
	Aqueous extract	Gastric adenocarcinoma (AGS) cells and normal intestinal cells (INT-407)	Reducing the potential of the mitochondrial membrane Bax/Bcl-2, Up-regulating cytochrome C	(154)
	Ethanolic extract	Mouse xenograft model of hepatoma Huh-7 cells	Interaction with the Wnt pathway co-receptor LRP6 on the cell membrane	(155)
	Aqueous garlic extract with lemon extract	BALB/c mice xenograft model of breast cancer EMT6/P cells	Inhibition of the expression of vascular endothelial growth factor, increasing interferon-γ, IL-2, and IL-4 levels	(156)
Antimicrobial activity	Ethanolic bulb extract	*Staphylococcus aureus, Escherichia coli, Pseudomonas aeruginosa, Bacillus cereus, Aspergillus versicolor*, and *Penicillium citrinum, P. expansum*	Increase bacterial growth inhibition	(157)
	Aged garlic bulb extract	*Burkholderia cepacia*	Increase bacterial growth inhibition	(158)
	Ethanolic bulb extract	*Bacillus subtilis, B. megaterium, B. polymyxa, B. sphaericus, Staphylococcus aureus, Escherichia coli, Penicillium oxalicum, Aspergillus flavus, A. luchuensis, Rhizopus stolonifer, Scopulariopsis* sp. and *Mucor* sp.	Increase bacterial growth inhibition	(159)
	Ethanolic bulb extract	*Staphylococcus aureus, Pseudomonas aeruginosa, Escherichia coli*	Increase bacterial growth inhibition	(31)
	Aqueous bulb extract	*Bacillus subtilis, Staphylococcus aureus, Escherichia coli, Klebsiella pneumoniae, Candida albicans*	Increase bacterial growth inhibition	(6)
	Garlic oil	*Penicillium funiculosum*	Destroying the cell structure, leading to the leakage of cytoplasm and macromolecules, inhibiting the bacterial growth	(32)
	Methanolic and water extracts of clove	*Bacillus cereus, Listeria monocytogenes, Staphylococcus aureus, Pseudomonas aeruginosa, Escherichia coli, Candida albicans, Rhodotorula mucilaginosa*	Inhibiting the bacterial growth	(33)
	Garlic clove oil	*Staphylococcus aureus, Escherichia coli, Pseudomonas aeruginosa, Salmonella typhi, Candida albicans*	Inhibiting the bacterial growth	(160)
Antidiabetic activity	Ethanolic extract	Streptozotocin and alloxan-induced diabetic mice and rabbits	Increased plasma insulin level, decreased plasma glucose levels, glucose-induced insulin secretion on the pancreas	(34)
	Aged garlic extract	Streptozotocin (STZ)-induced diabetic rats	Decreased blood glucose, increased serum insulin, accumulate insulin-glucose pathway	(35)
	Garlic pill	Prediabetic pregnant women	Reduced fasting blood sugar (FBS) level, prediabetic symptoms, and diastolic blood pressure	(36)

### Biological activity of plant extracts

#### Antioxidant activity

According to research, *A. sativum* possesses high antioxidant properties. A study compared the antioxidant qualities of raw and cooked cloves, finding that uncooked garlic has an intense antioxidant potential, while prepared ones have extensive antioxidant effects due to -carotene decolorization (11). Oxygen radical absorption capacity (ORAC) and DPPH experiments with HT22 rodent hippocampus cell culture revealed that the ethanolic infusion of garlic seedlings had antioxidant properties by DPPH scavenging activity and suppression of ROS (12). AG or aged garlic, as well as NG or non-aged garlic by-products that were extracted with distilled water, ethyl alcohol, or dichloromethane, showed distinct antioxidant effects as measured by radical scavenging tests ABTS, DPHH and H_2_O_2_, total Fe^3+^ reducing antioxidant power (FRAP) assay and Fe^2+^ chelating experiment (13). Unlike MCG or multi clove garlic isolates, SCG or single clove garlic ones exhibited a substantial increase in scavenging activity in the DPPH test and superoxide radicals. SCG is more resistant to CCl4-induced hepatotoxicity than MCG, and it may be an effective substitute drug for severe oxidative hepatotoxicity (14).

#### Hepatoprotective activity

The liver-protecting potential of fermented garlic extracts produced by lactic acid bacteria toward acetaminophen (AAP) resulted in acute liver damage in rodents was discovered in a study. Bacteria suppress AAP-induced cell death in hepatocytes by inhibiting the MAPK phosphorylation route and down-regulating p53, which is involved in liver autophagy, and by cytoplasmic redox command, as evidenced by reduction of oxidative stress, glutathione and ATP exhaustion, and antioxidant enzyme actions (15). Further analysis revealed that alcohol administration increased ROS/RNS generation in various animals. It depleted liver antioxidant status, decreased liver glutathione (GSH) concentrations, and decreased superoxide dismutase activity (SOD). Only garlic compounds can shield the liver from ethanol-induced peroxidation, as evidenced by a decrease in the marker of oxidative damage (malondialdehyde, MDA) and recovery of hepatoprotective action (16). The consequences of aqueous Garlic bulb isolate on alloxan-induced plasma advancements in liver enzymes and urinary metabolic indicators in Wistar rats were examined. The liver enzymes alanine transaminase, aspartate transaminase, and alkaline phosphatase have been associated with the leakage of cytoplasm from hepatocytes into the circulation as a condition of hepatotoxicity. The result showed that when compared to control, the plasma levels of all the parameters evaluated in the rats given garlic [urea, creatinine, albumin, aspartate transaminase (AST), alanine transaminase (ALT), and alkaline phosphate (ALP)] were unaltered (17). In the livers of albino rodents, Kaur and Sharma (18) found that ethanol extracts of garlic and ascorbic acid mixtures have hepatoprotective action toward Cd damage. Cadmium administered mice had a substantial increase in the amount of malignant growth, such as multinucleated nuclei and gigantic cells, which was reversed by administering ascorbic acid and ethanolic extract, which provided protection against the toxicity. Garlic also show hepatoprotective properties due to its organosulfur compounds like diallyldisulfide (DDS) and diallyltrisulfides (DTS), which have antioxidant and detoxifying properties. These compounds can reduce Cd-induced oxidative stress below the threshold level, produce preconditioning effects by activating survival signals, and activate DNA repair systems by reducing the binding of Cd with DNA (18).

#### Anti-inflammatory activity

In lipopolysaccharide treated J774A.1 macrophages (19) probed the anti-inflammatory attributes of *Allium* 14-kDa protein, something that impeded inflammatory agents such as nitric oxide (NO), prostaglandin E (PGE), TNF-, and interleukin (IL)-1 by impairing the nuclear factor-kappa B (NF-kB) signaling route (19). Morihara et al. (20) discovered that AG extract has a significant anti-inflammatory action and can assist to inhibit inflammatory responses in mouse models with apolipoprotein E knockout (ApoE-KO). In hepatocytes, AG treatment decreased TNF constructions, a prominent stimulus resulting in CRP synthesis, by 35%, downregulated interleukin-1 receptor-associated kinase 4 (IRAK4) by 60%, and increased adenosine monophosphate-activated protein kinase (AMPK) production (20). Ethyl linoleate (ELA), an essential fatty acid separated from garlic cloves, was demonstrated to suppress inducible nitric oxide synthase (iNOS) formed as a result of LPS treatment, transcription of cyclooxygenase-2 (COX-2) and the generation of pro-inflammatory cytokines. The inactivation of NF-B as well as the MAPKs and phosphorylation of the Akt circuits were responsible for this action. In RAW 264.7 cells, ELA-triggered heme oxygenase-1 (HO-1) regulates the reduction of LPS-induced NO and pro-inflammatory cytokine synthesis. In this study, the anti-inflammatory effects of ELA were established and ELA could be employed as a medical therapy to treat inflammation-related disorders (21). In BALB/c mice experiencing schistosomiasis, Metwally et al. (22) evaluated the possible anti-inflammatory effect of garlic extract and allicin against liver inflammatory indicators (*S. mansoni* infection). Both preventive and therapeutic injection of garlic extract or allicin into affected mice showed considerable immunomodulatory and anti-inflammatory benefits in this investigation. The immunohistochemical production of fibronectin and -SMA, as well as the transcription of inflammatory cytokines mRNA as indicators of liver fibrosis, indicate these consequences. Garlic significantly inhibited inflammatory cytokine expression, suggesting that the altered Th1/Th2 cytokine balance was the cause of the serum concentrations of liver fibrosis markers and proinflammatory cytokines. These markers were maintained by decreasing serum ALT and AST levels, granuloma size as well as the number of inflammatory cells, collagen fibers, and eggs in the granuloma (22).

#### Cardioprotective activity

Takashima et al. (23) found that the vasorelaxant impact of AG on the rat aorta had a substantial cardioprotective action in lowering arterial pressure. Endothelium-dependent vasorelaxation of the aorta is caused by AGE, which promotes vasodilation by increasing the synthesis of NO controlled by eNOS. L-arginine, a NOS precursor, was found to be one of the key elements of the AGE’s vasorelaxant action. Additionally, the source of AGE’s action to reduce arterial pressure in drug testing and *in vivo* testing remains unknown (23). On 22 total cholesterol tests (TC), 17 LDL cholesterol experiments (LDL-C), 18 HDL cholesterol tests (HDL-C), four fasting blood glucose tests (FBG), nine tribulations of systolic blood pressure (SBP) and 10 tests of diastolic blood pressure (DBP), *A. sativum* particle isolate was found to have cardioprotective activity. Garlic flour formulations were found to be a universal cardiac and circulatory tonic, lowering blood TC, LDL-C, HDL-C, FBG, SBP, and DBP (24). Another study looked at the effects of fermented garlic extract (FGE) on pulmonary arterial hypertension in rats given monocrotaline (MCT). In the right ventricular, MCT treatment increased weight, arterial stiffness, and atrial natriuretic peptide levels, although not in the left ventricle. FGE decreased these impacts, as well as pulmonary arteriole endothelial dysfunction and medial hypertrophy, pulmonary fibrosis produced by translations of PKG, MCT, and eNOS proteins in the lung, and increased translations of the VCAM-1 and MMP-9 proteins in the lung. FGE also inhibited an available guanylyl cyclase (sGC) inhibitor. The impact of FGE on MCT-induced heart attack in rats has been found to have cardioprotective effects, according to this research (25).

#### Anticancer activity

In rodents with 1,2-dimethylhydrazine (DMH)-induced tumorigenesis and multiplication of human colon tumor cells, Jikihara et al. (26) studied the anticancer effects of alcohol extracts of *A. sativum*. Pathological study shows that *A. sativum* extract can decrease adenocarcinoma and adenoma. Jikihara et al. (26) investigated the cytotoxic activity of *A. sativum* aqueous preparations in rodents with 1,2-dimethylhydrazine (DMH)-induced carcinogenesis and human colorectal tumor cell proliferation. Adenocarcinoma and adenoma can be reduced by isolate of *A. sativum*, according to histopathological research. Apoptosis was not induced by AGE, but it did slow down cell cycle progression by decreasing the expression of cyclin B1 and cdk1 in human colorectal cancer cells (26). The impacts of garlic extract on the growth of human breast cancer cell lines (MCF-7), prostate (PC-3), liver (Hep-G2), and colon (Caco-2), as well as mice macrophage cells, were investigated in a study (TIB-71). Exposure of Hep-G2, MCF-7, TIB-71, and PC-3 cells with crude garlic excerpt decreased cell proliferation by 80–90%, but only 40–55% in Caco-2 cells. It also caused growth arrest and a four-fold increase in caspase activation (apoptosis) in PC-3 cells. According to this research, crude garlic powder contains reactive fatty components and could be employed as a chemoprevention (27). A study examined the antitumor properties of diallyl trisulfide (DATS). *In vitro* and *in vivo* treatment of patient gastric cancer cell SGC-7901 using a flavonoid molecule produced from *A. sativum* isolate. Treatment with DATS hindered SGC-7901 cell growth by inducing apoptosis and amplifying the MAPK route by phosphorylating JNK, ERK, and p38. It also inhibited invasiveness by attenuating the utterances of the MMP9 and E cadherin proteins, induce cell death and amplifies the MAPK route by initiating JNK, ERK, and p38. Furthermore, DATS therapy increased cytokine release, such as TNF- and IFN-, which promoted the inflammatory system of the host in cancer care (28).

##### Gastric cancer chemoprevention by allicin

Allicin, a component of *A. sativum*, was found to be effective against stomach cancer, the fifth most common cancer in the world. For a long time, the mode of action was unknown. Allicin functions by halting the cell cycle during the G2/M phase and apoptosis in gastric tumor cells, while normal stomach cells remain unaffected, according to numerous investigations. Furthermore, even in the scientific results, allicin-mediated inhibition of stomach tumorigenesis can be seen. TGF-alpha (TGF-) and its receptor epidermal growth factor receptor (EGFR) are both inhibited by allicin (EGFR). This causes cyclin E and cyclin D1 to be down-regulated, which can cause cells in the G2 phase to enter the M phase. Allicin can cause DNA damage by causing reactive oxygen species (ROS), resulting in phosphorylated P53 and P21 proteins. P21 then inhibits the cyclin-dependent kinase 4/6 (CDK4/6)-cyclin D complex, causing the cell to enter G2/M arrest.

**FIGURE 3:**
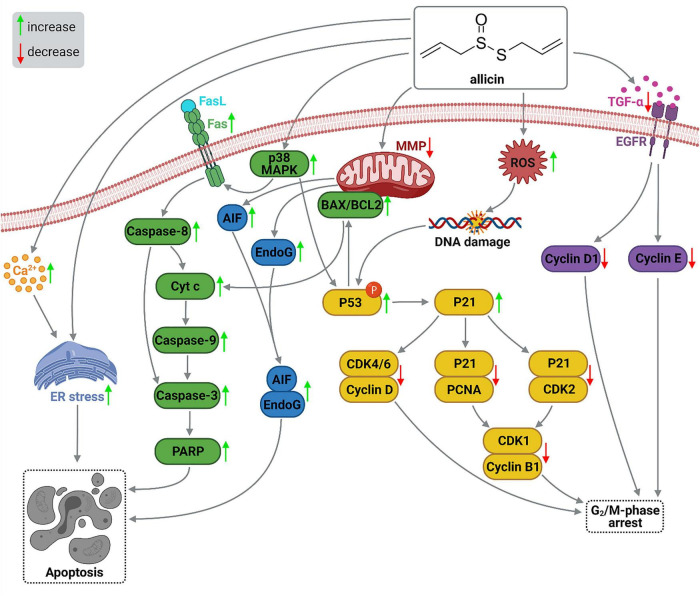
Allicin-mediated apoptosis signaling pathways in gastric cancer cells. AIF, apoptosis induce factor; BAX, BCL2-associated X protein; BCL2, B-cell lymphoma 2 protein; CDK, cyclin-dependent kinase; Cyt c, cytochrome c; EGFR, epidermal growth factor receptor; EndoG, endonuclease G; ER, endoplasmic reticulum; Fas, apoptosis antigen 1 protein; FasL, Fas receptor ligand; MAPK, mitogen-activated protein kinase; MMP, mitochondrial membrane potential; PARP, poly ADP-ribose polymerase; PCNA, proliferating cell nuclear antigen; ROS, reactive oxygen species; TGF-α, transforming growth factor alpha (29). This figure was prepared with BioRender.com.

Furthermore, the P21 polypeptide can block the P21-CDK2 and P21-proliferating cell nuclear antigen (PCNA) assemblages, causing the CDK1-cyclin B1 complex to diminish and the G2/M cell stage to be arrested. The particle’s lipid-soluble nature enables it to simply transfer across cellular barriers. Allicin significantly lowers the potential of the exterior mitochondrial membrane. Elevation in the proportion of pro-apoptotic polypeptide associated with BCL2 (BAX) to anti-apoptotic protein of B cell lymphoma 2 (BCL2) caused by allicin may promote cell death *via* the mitochondrial route. A greater BAX/BCL2 ratio causes cytochrome c to be released into the cytoplasm, accompanied by caspase-9 involvement. First, caspase-3 is triggered which results in poly ADP-ribose polymerase (PARP) and apoptosis-inducing polymerase (AIP) activation. Intrinsic stimulation of p38 mitogen-activated kinase or p38 MAPK activity was greater in allicin-induced gastric tumor cells. The synthesis of the Fas membrane-spanning peptide and P53 may be increased if p38 MAPK is increased. The Fas protein is part of the tumor necrosis factor or TNF receptor superfamily and contains 3 domains (extracellular, cytoplasmic, and transmembrane). The association of Fas’ cysteine-rich signal peptide with the Fas ligand (FasL) is a critical regulator of tumor growth (29). Caspase-8 is activated as a result of the interaction and can stimulate two separate apoptosis mechanisms. Caspases-8 trigger caspase-3 and caspase-7 in the mitochondrial independent route to trigger apoptosis. Caspase-8 is responsible for the liberation of Cyt c from mitochondria in the mitochondrial-dependent route (30). Allicin can also result in cell death through a process devoid of caspases. In this route, mitochondria generate apoptosis effectors such as apoptosis-inducing factor (AIF) and endonuclease G. (EndoG) which is the coactivator of AIF. EndoG and AIF are then teleported to the nuclei, where they lead to cell death through breaks in the nuclear material strands. Furthermore, allicin can cause apoptotic cell death by boosting unbound Ca^2+^ cation concentrations and inducing endoplasmic reticulum (ER) strain (Figure 3) (29).

#### Antimicrobial activity

The antibacterial activities of *Allium sativum* are extensive. Garlic essential oil [rich in diallyl monosulfide, diallyl disulfide (DADS), diallyl trisulfide, and diallyl tetrasulfide] extracted from raw bulbs had a good antibacterial action against *Pseudomonas aeruginosa, Staphylococcus aureus*, and *Escherichia coli*. The results suggest that the presence of the allyl group is essential for the antimicrobial property of such sulfide derivatives found in *Allium* (31). Additional studies have demonstrated that watery bulb infusion has high antibacterial efficacy against *Bacillus subtilis, Staphylococcus aureus, Klebsiella pneumoniae, Escherichia coli*, and *Candida albicans* due to disfunction in phospholipid bilayer synthesis by the activity of allicin (6). Garlic essential oil suppresses the fungal pathogen *Penicillium funiculosum*, presumably by infiltrating membranes and compartments, disrupting the cytoskeleton, and causing cytoplasmic and biomolecules leaks. In addition, proteomic analysis revealed the ability of garlic oil to increase or decrease the expression of few major proteins in relation to physiological metabolism (32). The antimicrobial effect of Australian garlic methanol and aqueous clove formulations was also described on *Candida albicans*, *Bacillus cereus, Escherichia coli, Staphylococcus aureus, Listeria monocytogenes, Pseudomonas aeruginosa*, and *Rhodotorula mucilaginosa*. The identification and quantification of pharmacologically potent compounds was done using ultra-high performance liquid chromatography with mass spectrometry and photodiode array detection (UHPLC-PDA-MS) and a correlation assessment was performed between the compounds and antioxidant and antimicrobial properties (33).

#### Antidiabetic activity

According to a review article, due to enhanced insulin-like efficacy, the antidiabetic behavior of *A. sativum* ethyl ether extract (at 0.25 mg/kg) was investigated in alloxan-induced diabetic rodents. The parietal cells of the pancreas are stimulated by ingestion of ethanol excerpt, pulp, and oil. Allicin boosted liver metabolism and pancreatic β cell insulin response (34). Another investigation found that 3 progressive doses of aged AE extracts have hypoglycemic activities in rats treated with streptozotocin. Diabetic rats were divided into two groups: control diabetics (CD), which served as negative control, and AGE-treated diabetics (AGE-D). AGE-Ds were classified into 3 groups and given AGE at 600, 300, and 100 mg/kg every day for a period of 2 months, as well as a normal control group (CN) which was the positive control for comparison. CD rodents had higher sugar levels (almost four times), twice the amount of serum cholesterol, triglycerides, and erythrocyte glycosylated hemoglobin, and thrice the amount of kidney and liver fatty acid oxidation, compared to the CN cohort. In the streptozotocin-treated diabetic group, the findings revealed that AGE had a dosage-dependent mitigative effect on hyperglycemia markers (35). In prediabetic expectant mothers, Faroughi et al. (36) evaluated the effects of the *A. sativum* tablet on the primary outcomes of fasting blood sugar or FBS and the recurrence of signs of prediabetes, as well as secondary outcomes such as hypertension, infant anthropometric parameters, and delivery method. Throughout 2015–2016, 49 mothers with prediabetes at 24–28 weeks of gestation were included in the triple-blind, randomly selected controlled study in Tabriz, Iran. The average concentration of FBS in the garlic-treated cohort reduced from 106.6 (11.1) mg/dL before therapy to 83.6 (6.3) mg/dL after 4 weeks and 79.4 (6.1) mg/dL 8 weeks later, according to the findings. The garlic drug also resulted in a substantial reduction in pre-diabetic signs at 4 weeks after therapy and diastolic arterial tension at 4 and 8 weeks. There was no substantial difference in systolic pressure between the cohorts at 4 and 8 weeks after treatment. As a result, the *A. sativum* tablet reduced FBS levels, prediabetes indicators, and diastolic BP in this study (36).

#### Antiviral activity

**FIGURE 4:**
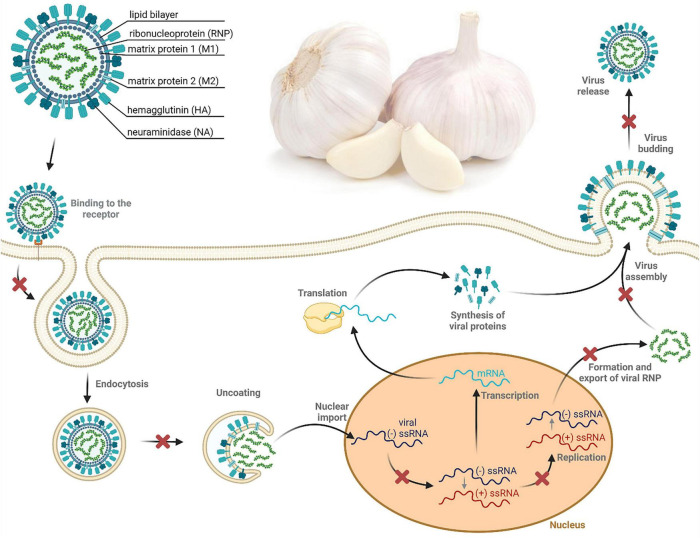
Proposed antiviral effects of *A. sativum* against (–) ssRNA viruses. This figure depicts the life cycle steps of influenza A with possible inhibition (shown as a red X-shaped symbol) mediated by garlic extracts or its organosulfur constituents. As shown in the figure, *A. sativum* bears potential for the prevention of viral penetration into the host membrane, virus uncoating associated with the release of viral genetic material into the cytoplasm, conversion of viral (–) ssRNA to viral (+) ssRNA, replication of the viral RNA, virus assembly and release (3). Figure made using BioRender.com.

*A. sativum* has long been traditionally used to cure a variety of viral illnesses. Garlic isolates or compounds have been shown to have antiviral action in several investigations (Figure 4). Extracts or isolates include chemical constituents that can attack different stages of the viral life cycle. Prevention of viral infection can be as simple as inhibiting the virus’s admission stage. Both encapsulated and non-enveloped pathogens are destroyed by this technique. *A. sativum* and its potent organosulfur components have been shown to promote antiviral activity by interacting with components on the exterior of the virion. This results in partial inhibition or complete blocking of the virus entry step (3). The aqueous extract of *A. sativum* expressed an effective inhibition of viral infection of influenza A subtype H1N1 with EC_50_ = 0.01 mg/ml in the Madin-Darby canine kidney (MDCK) cell line (37). The exact value of EC_50_ (0.01 mg/ml) was observed against Measles morbillivirus in Vero cells treated with gold nanoparticles containing aqueous extracts of *A. sativum* because it causes an obstruction of the viral receptors, stopping cell adsorption and the start of viral infection in the host cell (38). Furthermore, garlic extracts inhibited the binding of Newcastle disease virus (NDV) to chick embryo cell receptors (39). Quercetin, a flavonoid commonly present in fruits and vegetables, including *A. sativum*, possesses antiviral properties against influenza virus and enterovirus by affecting viral attachment to the surface of the host cell. Furthermore, organosulfur compounds in garlic such as allicin, ajoene, and diallyl trisulfide play an important role in the antiviral properties of garlic. These substances can prevent a virus from attaching to a host cell, change how the viral genome is translated in the host cell, and affect viral RNA polymerase, which is required for viral replication. They can also prevent the viral process that changes the host cell’s signaling pathway, and prevent viral multiplication (40). Even if the virus enters the host cell, there are still various steps suitable for its inhibition. One of these represents inhibition in the viral replication step. Replication may occur in the cytoplasm or in the nucleus of the host cell. In the current history, numerous investigations have proven the ability of *A. sativum* to prevent viral proliferation. The methanolic and ethanolic garlic extracts revealed inhibition of viral RNA polymerase and nucleoprotein synthesis activity against the influenza A (H1N1) pdm09 virus. This property may be attributed to their ability to block viral attachment and to suppress viral hemagglutinin (HA) (41). Furthermore, the study evaluating the influence of aqueous garlic extracts on avian infectious bronchitis virus (IBV), a coronavirus that infects birds of the Coronaviridae family, suggested the potential of *A. sativum* to inhibit the viral replication step (42). The members of the retrovirus family utilize reverse transcriptase (RT) to convert viral RNA into DNA. As a result, RT suppression is a major clinical strategy in the treatment of retrovirus transmission (3). The hexane extracts of *A. sativum* expressed a powerful activity against the RT of human immunodeficiency virus 1 (HIV-1) with an IC_50_ of approximately 64.08 ± 1.09 μg/mL (43). Furthermore, allicin was evaluated in the study to relieve immune dysfunction caused by the reticuloendotheliosis virus (REV). The study suggests that allicin downregulated the ERK/MAPK signaling pathway resulting in inactivation of REV replication (3, 44).

The World Health Organization (WHO) proclaimed a worldwide pandemic of COVID-19 on March 11th, 2020, triggered by SARS-CoV-2. Although various studies have been reported for the treatment of COVID-19, an effective remedy is still missing. However, several herbal or pharmaceutical drugs have been reported with possible beneficial effects on COVID-19. One of them is *A. sativum*. As mentioned above, aqueous garlic extracts possess an inhibitory effect against IBV of the Coronaviridae family. Therefore, it is assumed that it may also be beneficial against other members of the virus family, including SARS-CoV-2. Furthermore, some constituents of *A. sativum* seem to be helpful in reducing viral replication. Quercetin, a flavonoid present in garlic, bears the potential to inhibit the main protease of SARS-CoV-2 (M^pro^), a crucial antiviral target (45). As reported before, quercetin-3-β-galactoside was a suppressor of SARS-CoV-1 M^pro^ with IC_50_ = 42.79 ± 4.97 μM (46). Furthermore, there is a high structural similarity of M^pro^ between SARS-CoV-1 and 2 (∼96%). *In silico* molecular docking of organosulfur compounds in *A. sativum* revealed an inhibitory potential of alliin against M^pro^ SARS-CoV-2. The results suggested that alliin has potential antiviral activity against COVID-19 by binding to M^pro^ of SARS-CoV-2 (47). On the other hand, after the coronavirus outbreak, various hoaxes have been posted on the Internet about preventing COVID-19. Some of them argued that: “eating garlic prevents from COVID-19” (48). However, the WHO subsequently declared that there is no indication that garlic consumption has safeguarded patients from the novel coronavirus in the recent outbreak. Indeed, to date, no studies have been reported to confirm the prevention of *A. sativum* against COVID-19. However, there are no doubts about the beneficial effects of garlic on human health. In addition, studies recently reported confirming that despite garlic’s inability to prevent COVID-19, its antiviral activity can play a crucial role in alleviating patients suffering from COVID-19.

## Toxicological studies

Many investigations have been conducted on the toxicity assessment of *A. sativum* in laboratory animals. According to OECD standards 423, research explained the acute toxicity of A. sativum ethyl acetate concentrates and then determined the LD50 to demonstrate the acceptability of the extract in rodents (49). To test cytotoxic activity, all 3 animals in each set received ethanolic extract of *A. sativum* in a solitary administration of 300, 2,000, and 5,000 mg/kg of body weight. For each treatment, effects on body mass, skin tone, salivation, cornea, mucous system, drowsiness, tremor, unconsciousness, spasm, malaise, diarrhea, and death were observed after 30 min, 4 h, 24 h, 48 h, 1 week, and 2 weeks (49). Sections of the lungs, spleen, heart, kidneys, liver, and intestines were viewed during gross postmortem and microscopy using standard forensic methods. The rodents did not die as a consequence of the dose increase and there was no significant change in any of the criteria used for general post mortem and histopathological alteration. As a result, the extract is fine for humans to consume (49). Another investigation looked at whether *A. sativum* preparations had a suppressive effect on lead nitrate poisoning. Their potential to eliminate free radicals and avoid GSH deficiency could describe the function of garlic organosulfur compounds in reducing Pb(NO_3_)_2_ poisoning (50). Total erythrocyte count, hemoglobin concentration, total leukocyte count, lymphocyte and monocyte percentage all decreased significantly after chronic consumption of lead nitrate. In the renal and central nervous system, the intake of lead nitrate resulted in an increase in the levels of reactive components of thiobarbituric acid, as well as a reduction in glutathione peroxidase levels and antioxidant molecules such as superoxide dismutase and catalase. Nitrate aids lower peptide levels, whereas cholesterol and lead burden increase exponentially in lead-exposed mice, resulting in decreased plaque count, immunoglobulin level, phagocytic index, and macrophage viability (50). Orally ingested extracts of *A. sativum* by exposed groups to Pb(NO_3_)_2_, on the other hand, may be a preventive strategy against heavy metal toxicity (50). In cultivated Vero cells, Abid-Essefi et al. (51) explored the role of an aqueous extract of *A. sativum* (AEA) vs. zearalenone (ZEN) mediated lethality, reactive oxygen species (ROS), and genomic instability. The findings revealed that ZEN caused several harmful consequences and major changes in Vero cells, which were regulated through the oxidative stress system. Administration with AEA (250 μg/ml) in combination with ZEN resulted in a considerable decrease in ZEN-induced impairments for each marker evaluated, as well as a notable fall in DNA disintegration. As a result, AEA can assist guards against ZEN risks. The great potential of AEA to compensate for the inflammatory process generated by ZEN is probably responsible for its preventive action (51). Many research has suggested that garlic could be used as a food additive for people who are not protected from ecological toxins. Irkin et al. (52) studied the effects of dietary garlic granules on young European sea bass (*Dicentrarchus labrax*). For 2 months, the fish received enhanced meals with *A. sativum* powder at concentrations of 0 (control), 2 percent, 4 percent, or 6 percent. The RBC count, hemoglobin level, amount of hemoglobin and mean corpuscular hemoglobin in treated fish at dietary incorporation stages of 4 and 6% were considerably less than baseline data after this study. In contrast to the reference cohort, serum sugar levels in fish supplemented with garlic powder were considerably lower, and cholesterol values in the treatment groups of 6 and 2% were smaller than those of the control group. As a consequence, it is recommended that garlic powder fortification in the diets of juvenile fish not surpass 2%. As a result, this experiment concluded that nutritional garlic powder is critical to improving the physiological and hematological state of immune improvements in young sea bass (52).

## Clinical studies

*A. sativum* preparations are used in drug testing to alleviate a variety of human disorders. The bulbs have been used successfully as nutrition and treatment in human societies since antiquity. The possible hypoglycemic benefits of *A. sativum* in patients with type 2 diabetes were examined by Ashraf et al. (53). The purpose of this study was to compare the impact of garlic pills exposure to routine anti-diabetic analyzes on glucose levels in 60 confirmed type 2 diabetic patients with fasting blood glucose levels above 126 mg/dl. Participants were placed into two categories. Serum triglycerides and fasting blood glucose were measured at weeks 0, 12, and 24. Compared to group 2, group 1 reported a substantial reduction in fasting blood glucose at week 24. A mixture of *A. sativum* with traditional anti-diabetic medication improved glucose control and hypocholesterolemic action, according to this research. Garlic’s antilipidemic effects are thought to be caused by allicin’s ability to inhibit hydroxymethylglutaryl-CoA reductase, which lowers LDL-C levels as well as total cholesterol (53). Sukandar et al. (54) investigated the efficacy and tolerability of combining curcumin and *A. sativum* powder as an antidiabetic and hypocholesterolemic treatment for type 2 diabetes-dyslipidemia. 3 replicates were calculated which were 1.2, 1.6, and 2.4 g, respectively. The garlic-curcuma mixture was observed to lower glucose concentration and HbA1C while also improving dyslipidemia. The 2.4 g dose was found to reduce fasting glucose, glucose levels two hours after lunch, lipid profile, HbA1C, LDLs, cholesterol, and body mass index and increased HDLs greater than the other two prescriptions (54). The benefits of *A. sativum* supplementation on serum concentrations of certain markers of inflammation, somatic manifestations, and exhaustion in females with chronic rheumatoid arthritis or RA were studied by Moosavian et al. (55). In this investigation, a double-blind, placebo-controlled, randomized experiment was used. A group of 70 women with RA were randomly classified into two parts for 8 weeks, where the treatment group received 1,000 mg of *A. sativum* and the control team received a placebo (55). After therapy, the bioavailability of the C-reactive peptide (CRP) and TNF (TNF-a) was found to be considerably lower in the garlic cohort compared to the placebo category. Additionally, relative to the placebo group, pain severity, sensitive joint tally, disease activity score, and exhaustion were considerably lower, and the number of enlarged joints was markedly smaller in the garlic unit, but nothing of this type was demonstrated in the placebo. The result showed that garlic improved the inflammatory mediators like serum level of CRP and TNF-a, though there was no change in erythrocyte sedimentation rate (ESR) (55).

## Discussion

*A. sativum* was found to have a spectrum of therapeutic benefits in this investigation, including antibacterial, cardioprotective, anti-inflammatory, antitumor, and antispasmodic activities, among others. The most essential components of *A. sativum* are organosulfur molecules, which are responsible for most of their medicinal uses. allyl methyl sulfide, Allicin, ajoene, and DTS are the primary physiologically active chemicals responsible for the antibacterial, antifungal, antiviral, and antiprotozoal effects of *A. sativum*, respectively (1). The main ingredient of garlic, allicin, can cause stomach disturbances, particularly when taken in large doses. Most cardiac investigations have focused on triglycerides, LDL, cholesterol level, HDL, and arteriosclerosis (56). According to suggestions, *A. sativum* lowers LDL and triglyceride concentrations in people with dyslipidemia (56). Consumption of *A. sativum* reduces triglyceride levels, which may help slow the progression of hypertension. In addition to considerable studies, its antidiabetic, antibacterial, anti-inflammatory, and antioxidant effects have been linked in various models. Several investigations have looked into the antitumor and anticarcinogenic properties of *A. sativum* and its components *in vitro* and in animal experiments. According to the findings, *A. sativum* encompasses a set of crucially significant compounds with anticancer and chemopreventive activities. Garlic includes various physiologically and pharmacologically important components, according to clinical and scientific research. For cardiac, hyperglycemia, rheumatism, gastrointestinal disorders, colitis, liver disorders, bloating, diarrhea, bronchitis, sensory loss, hypertension, asthma, and other ailments, these are vital for health. Several investigations are underway throughout the world to make efficient and unscented garlic formulations and identify bioactive constituents that may have medicinal value (57–59). However, the extraction, production, molecular, physicochemical and structural properties and structure–activity relationships of garlic compounds (60–64) are beyond the scope of the present review. The metabolic processes of ingested garlic constituents (as food or supplements), their mechanisms of action in healthy humans have not been detailed well. Besides structure-bioactivity relationships remain unclear in many pharmacological attributes.

## Conclusion

For centuries, *A. sativum* has been widely consumed as a culinary and therapeutic herb as protective and curative agents. It contains essential minerals, vitamins, and protein and is widely used as a spice or condiment in continental cooking. In addition, this plant has various potential pharmacological activities against various diseases and disorders owing to its potent antioxidative, anti-inflammatory and immunomodulatory properties. Based on preclinical studies, *A. sativum* compounds especially the sulfur-containing compounds, some flavonoids and polyphenols may help treat certain human conditions, particularly those related to cancer and cardiovascular disease. The roles of *A. sativum* and the preparations of *A. sativum* in human health will help to benefit from additional well-designed human studies and various human diseases that carefully characterize the garlic interferences used and examine possible differential effects in several human populations. This research also looked at the key ingredients of the plant, such as sulfur-containing chemicals. Many significant advances have been found in the phytochemistry and related medicinal properties of *A. sativum*; data regarding clinical aspects and impact on human health-related properties are acceptable. Therefore, it is inferred that *A. sativum* is a fantastic seasoning that needs to be handled with prudence to fully benefit from its immense medicinal benefits, taking into account that misapplication may result in unintended consequences.

## Author contributions

CT, TD, MG, PB, DS, PO, NJ, MK, Radha, JP, JMP, and AD contributed to the concept, literature mining, writing, and methodology of the review, provided critical feedback, and critically revised the manuscript. CT came up with the study idea, planned and designed the review structure, wrote the first draft of the manuscript, prepared the table and figures, and had the right to list his name first in his CV, any other scientific documents, and scientific profile. TD, MG, PB, and DS contributed to writing review and editing, arranged references, and revised the table and figures. PO contributed to study idea, writing-review and editing, prepared the table and figures, arranged the references, and acquired funding. NJ, MK, and Radha participated in review structure, writing-review and editing, completed the critical revision of the entire manuscript, response, data validation, and suggestions. JP completed the critical revision of the entire manuscript, formal interpretation, and supervised the drafting process of the review, response, final draft, and resources. JMP contributed to manuscript revision, formal interpretation, supervision, response, resources, project administration, and funding acquisition. AD came up with the study idea, revised the review structure, suggestions, completed the critical revision of the entire manuscript, formal interpretation, and supervised the drafting process of the review, resources, and final draft. All authors have read and approved the final version of the manuscript for submission to this journal and contributed to the writing or revision of the final manuscript.

## Footnotes

1
https://www.drugs.com/npp/garlic.html
