# Corrigendum: Traditional uses, phytochemistry, pharmacology and toxicology of garlic (*Allium sativum*), a storehouse of diverse phytochemicals: a review of research from the last decade focusing on health and nutritional implications

**DOI:** 10.3389/fnut.2024.1430483

**Published:** 2024-07-29

**Authors:** Champa Keeya Tudu, Tusheema Dutta, Mimosa Ghorai, Protha Biswas, Dipu Samanta, Patrik Oleksak, Niraj Kumar Jha, Manoj Kumar, Radha, Jarosław Proćków, José M. Pérez de laLastra, Abhijit Dey

**Affiliations:** ^1^Department of Life Sciences, Presidency University, Kolkata, India; ^2^Department of Botany, Dr. Kanailal Bhattacharyya College, Howrah, India; ^3^Department of Chemistry, Faculty of Science, University of Hradec Kralove, Hradec Kralove, Czechia; ^4^Department of Biotechnology, School of Engineering and Technology, Sharda University, Greater Noida, Uttar Pradesh, India; ^5^Department of Biotechnology, School of Applied and Life Sciences, Uttaranchal University, Dehradun, India; ^6^Department of Biotechnology Engineering and Food Technology, Chandigarh University, Mohali, India; ^7^Chemical and Biochemical Processing Division, ICAR-Central Institute for Research on Cotton Technology, Mumbai, India; ^8^School of Biological and Environmental Sciences, Shoolini University of Biotechnology and Management Sciences, Solan, India; ^9^Department of Plant Biology, Institute of Environmental Biology, Wrocław University of Environmental and Life Sciences, Kożuchowska, Poland; ^10^Biotechnology of Macromolecules, Instituto de Productos Naturales y Agrobiología, IPNA (CSIC). Avda, Astrofísico Francisco Sánchez, San Cristóbal de la Laguna, Spain

**Keywords:** *Allium sativum*, traditional uses, ethnobotany, phytochemistry, pharmacology, toxicology

A correction has been made to the **Biological activity of plant extracts**, “*Antidiabetic activity*.” In the published article, there was an error resulting from a discrepancy in the conversion of units and the reference to a research article instead of a review article.

This sentence previously stated:

“According to a research article, due to enhanced insulin-like efficacy, the antidiabetic behavior of *A. sativum* ethyl ether extract (at 0.0025 g/kg) was investigated in alloxan-induced diabetic rodents.”

The corrected sentence appears below:

“According to a review article, due to enhanced insulin-like efficacy, the antidiabetic behavior of *A. sativum* ethyl ether extract (at 0.25 mg/kg) was investigated in alloxan-induced diabetic rodents.”

A correction has been made to the **Toxicological studies**. In the published article, there was an error resulting from a discrepancy in the conversion of units and the reference to lean mass instead of body weight.

This sentence previously stated:

“To test cytotoxic activity, all 3 animals in each set received ethanolic extract of *A. sativum* in a solitary administration of 3, 20, and 50 g/kg of lean mass.”

The corrected sentence appears below:

“To test cytotoxic activity, all 3 animals in each set received ethanolic extract of *A. sativum* in a solitary administration of 300, 2,000, and 5,000 mg/kg of body weight.”

In the same section **Toxicological studies**, there was an error resulting from a discrepancy in the conversion of AEA units, the reference to more than one concentration used, and the reference to changes in the body rather than in the Vero cells.

This sentence previously stated:

“The findings revealed that ZEN caused several harmful consequences and major changes in the body, which were regulated through the oxidative stress system. Administration with the smallest amount of AEA (250 g/ml) in combination with ZEN resulted in a considerable decrease in ZEN-induced impairments for each marker evaluated, as well as a notable fall in DNA disintegration.”

The corrected sentence appears below:

“The findings revealed that ZEN caused several harmful consequences and major changes in Vero cells, which were regulated through the oxidative stress system. Administration with AEA (250 μg/ml) in combination with ZEN resulted in a considerable decrease in ZEN-induced impairments for each marker evaluated, as well as a notable fall in DNA disintegration.”

The authors apologize for these error and state that this does not change the scientific conclusions of the article in any way. The original article has been updated.

