# Supplementary material for: Traditional uses, phytochemistry, pharmacology and toxicology of garlic (Allium sativum), a storehouse of diverse phytochemicals: A review of research from the last decade focusing on health and nutritional implications
Source: Front Nutr. 2022 Oct 28;9:949554. doi: 10.3389/fnut.2022.929554 (PMC9650110; doi:10.3389/fnut.2022.929554)
Supplement: Supplementary file 1 [file Table_1.docx]

**Table S1.** Ethnobotanical uses of *A. sativum*

| **Plant parts** | **Place** | **Local name** | **Ethnic group** | **Ethnomedicinal use against/as** | **Method of preparation** | **Route of administration/dose** | **References** |
| --- | --- | --- | --- | --- | --- | --- | --- |
| Bulb | Upper Dibang Valley, Arunachal Pradesh, India | Chong | Adi, Monpa, Apatani, Nyishi | Stomach bloating | Paste | Oral | Devi et al., 2014 |
|  | Suva planina, south-east of Serbia | Beli luk | Shopi | Improved blood circulation, reducing triglycerides | Mixed with 3 chopped lemons and added to 1 l of hot water and left to sit for 12 hours | One cup is drunk a day (for 40 days) | Jarić et al., 2015 |
|  |  |  |  | Pneumonia | Mixed with honey | One teaspoon to be taken 3 times a day with warm water |  |
|  |  |  |  | Hair loss prevention | Mixed with rosemary leaves and added to isopropyl alcohol | Topical |  |
|  | Edo State,  Nigeria | Ayur | Eje riru,  Esagie no vba, Emiamudu, Aranoyokwiri, Obala mgbani enu | Hypertension | Added with soak leaves of guava and *Vernonia amygdalina* (Asteraceae), in water 5 day (maceration) | Half glass cup daily | Gbolade, 2012 |
|  |  |  |  | Hypertension | Mixed with bulbs of *Allium cepa* (Amaryllidaceae) and the rhizome of *Zingiber officinale* (Zingiberaceae), boiled in water (decoction) | One glass has taken twice daily |  |
|  | Alasehir district, Turkey | Sarmisak | Local people | Snakebite | Crushed | Topical | Ugulu, 2011 |
|  | South-Western Nigeria | Ayuu | Yoruba | Haemorrhoids | Cut *Anthocleista djalonensis* (Gentianaceae), *Aframomum melegueta* (Zingiberaceae), *Rauvolfia vomitoria* (Apocynaceae), *Allium ascolanicum* (Amaryllidaceae), *Syzygium aromaticum* (= *Eugenia aromatica,* Myrtaceae), *Acacia nilotica* (Fabaceae), *Dalbergiella welwitschii* (Fabaceae), *Monodora myristica* (Annonaceae) together and placed in a 4-litter keg and soaked with clean water and alcoholic beverages | Oral | Soladoye et al., 2010a |
|  | Ogun State, Nigeria | Ayu | Yoruba | Rheumatoid-arthritis | Mixed with *Cassia fistula* (Fabaceae) leaves and pure honey | Topical (cream) | Ogbole et al., 2010 |
|  | Ogun State, Nigeria | Alubosa ayun | Yoruba | Anticancer | Dried *Zingiber officinale, Curcuma longa* (= *C. domestica*, Zingiberaceae), *Tetrapleura tetraptera* (Fabaceae), *Crinum jagus* (Amaryllidaceae)*, Piper guineense* (Piperaceae) mixed with honey | One teaspoonful is taken 3 times daily | Soladoye et al., 2010b |
|  | Lower Eastern Province, Kenya | Kitunguua kinene | Kamba | Diabetes | A hot infusion of pounded bulb | Oral | Keter and Mutiso, 2012 |
|  | Chtouka Ait Baha and Tiznit (Western Anti-Atlas), Morocco | Tiskert /  Touma | Berber | Diabetes | Decoction | Oral | Barkaoui et al., 2017 |
|  | Niti Valley, Central Himalaya, India | Lashun | Bhotiya | Paralysis | Five bulbs are fried in 50ml of mustard oil and filtered | The oil is then rubbed on the infected part before sleeping | Phondani et al., 2017 |
|  |  |  |  | Carbuncles | Five bulbs are ground and mixed with 1g of salt and made into a paste | Topical |  |
|  | Kilte Awulaelo District, Tigray Region,  Ethiopia | Tsaeda  shingurti | Tigrigna | Wound, sore | Crush by mixing with leaves of *Dyschoriste radicans* (Acanthaceae) and *Lepidium sativum* (Brassicaceae) | Topical | Teklay et al., 2013 |
|  |  |  |  | Cough | Crush and eat it with honey | Oral |  |
|  |  |  |  | Paralysis | Crush | Rub on the body |  |
|  |  |  |  | Malaria | Crush and to take alone or by mixing it with seeds of *Lepidium sativum* | Oral |  |
|  |  |  |  | Itching, scabies | Crush by mixing with leaves of *Rumex nervosus* (Polygonaceae) and *Withania somnifera* (Solanaceae) and seeds of *Lepidium sativum*, soak it in water | Wash body |  |
|  | Ouémé, Southern Benin | Ayo | Goun, Yoruba | Hypertension | Decoction/infusion/maceration | Oral | Lagnika et al., 2016 |
|  | Central Region of Togo, Africa | Ayo | Tem tribe | Diabetes mellitus | Decoction with stem bark of *Khaya senegalensis* (Meliaceae) | Oral | Karou et al., 2011 |
|  |  |  |  | Hypertension | Crushed maceration and mixed with honey | Oral |  |
|  |  |  |  | Hypertension | Maceration with stem bark *Parkia biglobosa* (Fabaceae) | Oral |  |
|  |  |  |  | Hypertension | Powdered with leaves of *Lippia multiflora* (Verbenaceae) and *Stachytarpheta indica* (= *S. angustifolia*, Verbenaceae) | Oral |  |
|  | Chagharzai Valley,  Buner District, Pakistan | Ooga | local elderly people, Hakims | Heart diseases, asthma, whooping  cough | Decoction | Oral | Sher et al., 2011 |
|  | Samudrapur tahsil, Wardha district, Maharashtra, India | Garlic | Vaidus | Heat, pain reliever,  dysentery,  swelling, to stop  bleeding, cold,  lower down  blood sugar and blood pressure | The powder should be applied to the painful part; it is also used to stop bleeding; make turmeric, salt, and garlic in equal amount into a paste | Topical, oral | Shende et al., 2014 |
|  | Debre Libanos Wereda, Central Ethiopia | Nech shinkurt | Local people | Hypertension | Mixed with the *Rumex abyssinicus* (Polygonaceae) root powder and add this mixture into boiled water | Drink the hot decoction in a cup | Getanehand Girma, 2014 |
|  |  |  |  | Abdominal pain | Crush the bulb and mix with honey | Take a teaspoon each morning |  |
|  |  |  |  | Snakebite | Crushed | Topical |  |
|  | Gallies, Abbottabad, Northern Pakistan | Thoom | Jadoons, Karrlas, Dhunds,  Sardars | Asthma, whooping cough, respiratory disorders | Extract | Oral | Kayani et al., 2014 |
|  | Riverside of Navarra, Iberian Peninsula | Ajo | Local people | Chilblains | Direct application (fresh) | Topical | Calvo et al., 2011 |
|  |  |  |  | Rheumatism, arthritis | Juice with honey and  vinegar | Oral |  |
|  | Amaro Woreda, Ethiopia | NechshInkurte | Amharic, Oromiffa, Koorete | Influenza | Fresh bulbs (5–10) | Oral | Mesfin et al., 2014 |
|  |  |  |  | Headache,  chest pain,  malaria,  stomach ache | Bulbs (3–7) are chewed and swallowed | Oral |  |
|  | Oyo, Ogun, Osun state, Southwestern Nigeria | Ayuu | Yoruba | Female infertility | *Kigelia africana* (fruit, Bignoniaceae), *Tetrapleura tetraptera* (root, Fabaceae), *Aristolochia repens* (root, Aristolochiaceae) are boiled together in clean water for about 50 min | One small glass cup to be taken twice daily | Soladoye et al., 2014 |
|  | Abaya District,  Borana, Oromia, Ethiopia | Nechshinkurte | Guji Oromo | Bronchitis | Mixed with the crushed dry root of *Momordica foetida* (Cucurbitaceae) | Taken orally  before breakfast for three days | Bekele and Reddy, 2015 |
|  |  |  |  | Malaria | Mixed with the crushed seed of *Lepidium sativum* and honey | Taken orally for 5 days before breakfast |  |
|  |  |  |  | Malaria | Crushed fresh/dry leaves of *Croton macrostachyus* (Euphorbiaceae) and boiled together with water, then roasted with butter and left-over night outside | Taken orally in the morning |  |
|  | Tripura, India | Rasun, Risum | Tripuri, Jamatia, Reang, Noatia, Chakma, Bhil,  Bhutia, Garo, Chaimal, Halam, Khasia, Kuki, Lepcha, Lushai  Mag, Munda, Kaur, Orang, Santhal, Uchai | Heart problem, arthritis, colic pain, bronchitis | Fresh | Oral | Sen et al., 2011 |
|  |  |  |  | Skin disease | Paste | Topical |  |
|  | Horro  Gudurru Woredas, Western Ethiopia | Qullubbii adii | Oromo | Evil eye | Fresh/dried bark of *Stereospermum kunthianum* (Bignoniaceae), bark of  *Croton macrostachyus* (Euphorbiaceae), the root of *Cucumis ficifolius* (Cucurbitaceae), and the seed of *Capsicum frutescens* (Solanaceae) powdered together | Oral | Birhanu et al., 2015 |
|  | Zlatibor district, South-Western Serbia | Beli luk | Croatian,  Montenegrin | High blood pressure, high cholesterol level | Fresh | Oral | Šavikin et al., 2013 |
|  | Salija VDC, Parbat district, Central Nepal | Lasun | Magar | Gastritis | A few pieces of the roasted bulb | Oral | Thapa, 2012 |
|  |  |  |  | Skin boils | Paste | Topical |  |
|  | Bosnia and Herzegovina country, western  Balkan Peninsula | Bijeli luk | Bosniaks, Serbs, Croats | Cough, tooth ache, gingival  inflammation | Syrup | Oral | Šarić-Kundalić et al., 2010 |
|  |  |  |  | Increased  diuresis, chest ache, prophylaxis against  tuberculosis | Fresh | Oral |  |
|  |  |  |  | Urinary bladder stones | Decoction | Oral |  |
|  | Wayu Tuka District, Oromia  Regional State, West Ethiopia | Qullubbii adii | Oromo | Malaria | Taken with ‘injera’ and *Capsicum annuum* (Solanaceae) | Before eating breakfast | Megersa et al., 2013 |
|  | Akoko Region, Ondo-State, Nigeria | Ayuu | Lwaro Oka, Ayegunle Oka, Sumerin Oka Oba Akoko | Vaginal discharge, enhance sexual ability,  hormonal imbalance | *Senna alata* (Fabaceae), *Senna podocarpa* (Fabaceae), *Allium gomphrenoides* (= *A. ascalonium*, Amaryllidaceae) are kept in a closed container with hot water for 24 hours | One hundred ml is taken  orally every morning for 3 day | MK et al., 2016 |
|  | Arribes del  Duero, western Spain | Ajo | Galicians, Leonese | Chilblains, toothache | Fresh | Topical | González et al., 2010 |
|  | Dangme West district,  southern Ghana | Garlic | Dodowa, Ayikuma, Agomeda, Dawenya, Afienya, Prampram | Diabetes mellitus | Add to food | Oral | Asase and Yohonu, 2016 |
|  | Edremit Gulf,  Turkey | Sarımsak | Roman, Balkan | Antihypertensive | Dried | Oral | Polat and Satıl, 2012 |
|  |  |  |  | Cold, flu | Dried | Taken as a pill every day for 2–3 days |  |
|  |  |  |  | Carminative (for animal) | Pressing with milk | Drink one large cup.  two times a day |  |
|  | Dohanaga, Patnitala Upazilla,  Naogaon, Bangladesh | Rashunin | Santhal | Rheumatism | Juice | Topical | Rahman et al., 2014 |
|  | Gollak region, Kosovo, Southeastern Europe | Hudhera,  Hudra | Albanian | Warts | Rubbed on the warts | Topical | Mustafa et al., 2012a |
|  |  |  |  | Ear-ache | Juice applied into the ear | Topical |  |
|  |  |  |  | Anti-hypertensive | Fresh | Eaten |  |
|  |  |  |  | Disinfect the  intestine | Boiled in milk  (4–5 cloves) | Drink as tea |  |
|  | Woreda, southern Ethiopia | Tuma | Halaba | Stomach ache | Crushed and swallowed | Oral | Regassa et al., 2017 |
|  |  |  |  | Cold, malaria,  pneumonia | Crushed the bulb, add some spoons of honey | Orally in every morning |  |
|  | Oyo, Ogun, Ekiti, Lagos state, Southwestern Nigeria | Ayuu | Ijebu, Awori, Egun | Body cream | Juice extract | Topical | Fred-Jaiyesimi et al., 2015 |
|  | Loma district, Gena  Bosa District, Dawro Zone,  Southern Ethiopia | Tuumuwa | Kafficho, Bosat | Stomach ache,  rheumatism, snake bite  and snake repellant,  tonsillitis, typhoid, leech  expellant | Chewed, chopped,  pounded | Oral | Agize et al., 2013 |
|  | Kosovo, Southeastern Europe | Hudhra | Albanian | Improve blood  circulation  anti-diabetic  antibacterial  anti-hypertensive | Tincture | Oral | Mustafa et al., 2012b |
|  | Jimma Zone, Kersa woreda District, Oromia  Regional State, Southwestern Ethiopia | Qullubii adii | Oroma | Repel mosquitoes | Crushing and applying  the juice on the skin | Topical | Karunamoorthi and Hailu, 2014 |
|  | Minjar-Shenkora district,  Amhara Region, Ethiopia | Nechshink  Urt | Amhara, Oromo,  Argoba | Malaria | Eat with injera | Oral | Alemayehu et al., 2015 |
|  | Ogbomoso, Southwest Nigeria | Ayuu | Yoruba | Malaria | Added with leaves of Basil, *Capsicum* sp. (Solanaceae), *Citrus aurantiifolia* (Rutaceae), *Camellia sinensis* (Theaceae) and then boiled in local pot | A full glass cup is taken orally twice daily | Olorunnisola et al., 2013 |
|  | Paschim Medinipur district, West Bengal | Rosun | Santhals, Mundas, Lodhas, Bhumijs, Oraon Kherias | Snakebite | Paste | Oral | Sarkhel, 2014 |
|  | Navarra, Iberian Peninsula | Ajo | Basque | Chilblains,  boils, wounds,  warts | Direct application | Topical | Cavero et al., 2011 |
|  | Tata Province, Morocco | Thouma – Tiskrt | Amazigh,  Jews, Saharan, Arab-Muslim | Hypotensive, colds,  helminthiasis, depurative,  sterility | Decoction, raw | Oral | Abouri et al., 2012 |
|  |  |  |  | Snake and scorpion bite | Cataplasm | Topical |  |
|  |  |  |  | Antitoxic | Raw | Oral |  |
|  |  |  |  | Intestinal pains | Raw | Oral |  |
|  |  |  |  | Sty, skin infection,  antifungal | Boiled in oil | Topical |  |
|  | Chungtia village,  Nagaland, India | Lasung | Ao tribe | High blood pressure | Bulbs are kept in the mouth | Oral | Kichu et al., 2015 |
|  |  |  |  | Spider and snake bites | Paste | Topical |  |
|  | Pešter Plateau, Sandžak, South-Western  Serbia | Hundhura, Bjeli luk | Albanians, Serbs | Evil eye, eye  inflammations | Crushed | Topical | Pieroni et al., 2011 |
|  |  |  |  | Cold, hypertension, blood cleansing | Fresh | Oral |  |
|  | Ripollès district, eastern Pyrenees | All | Catalan | Antieczematous, antifungal | Direct use | Topical | Rigat et al., 2015 |
|  |  |  |  | External antiseptic | Embrocation | Topical |  |
|  |  |  |  | Pernio (chilblain) | Direct use | Topical |  |
|  |  |  |  | Resolvent,  vulnerary | Embrocation | Topical |  |
|  | Jos, Plateau State, Nigeria | Tafarmuwa | Hausa, Igbo, Yoruba | Common cold | Decoction | Oral | Ohemu et al., 2014 |
|  | Kembatta Tembaro (KT) Zone,  Southern Ethiopia | Tuma | Kembatta | Diarrhea | Decoction | Oral | Maryo et al., 2015 |
|  | Bishnupur district, Manipur, India | Satun | Chothe | Paralysis, rheumatic complaints, muscular pain, worm diseases, piles | Decoction | Oral | Purbashree et al., 2012 |
|  | Abbottabad district, North-West Frontier Province, Pakistan | Thom | Abbasies, Tareen, Jadon, Syeds, Mashwani, Tanolis, Awans | Earache | Fried in mustard oil | Topical | Abbasi et al., 2010 |
|  | Cachar district, Assam, India | Parunvar | Hmar tribe | Gastrointestinal problem | Fresh | Oral | Nath and Choudhury, 2010 |
|  | Kamranga village, Cachar district, Assam, India | Thingkh | Chirus | Pus | Paste | Applied on injuries | Singh et al., 2011 |
|  |  |  |  | Blood pressure | Cooked with vegetables | Oral |  |
|  |  |  |  | Paralysis | Paste with the seed of *Narthex asafoetida* (= *Ferula asafoetida*, Apiaceae) and butter | Massaged on body parts |  |
|  | Sub-Himalayan tracts, Uttarakhand, India | Ganda | Gujjar | Dysentery | Crushed bulbs are mixed with fodder or wheat husk | Oral | Gaur et al., 2010 |
|  | Malda district, West Bengal, India | Rosun | Santal, Oraon, Rajbanshi, Namasudre, Polia, Mundas, Malpaharias | Anthelmintic, dysentery | Crushed along with fodder | Oral | Saha et al., 2014 |
| Cloves | Upper Dibang Valley, Arunachal Pradesh, India | Chong | Adi, Monpa, Apatani, Nyishi | Cough, cold, chest congestion | Crushed into powder | Oral | Devi et al., 2014 |
|  | Poonch district, Azad Kashmir Pakistan | Thom | Tribal women | Toothache | The extract is made and cotton is dipped in it | This cotton is applied to the teeth | Shaheen et al., 2014 |
|  |  |  |  | Pimples | Crushed and powder | Topical |  |
|  |  |  |  | Weak nails | Slices | Rubbed on nails |  |
|  | Bellary district, Karnataka, India | Bellulli | Medara, Lambani, Korava, Budabudikae, Adavichencharu | Cough | Fresh with salt | Oral | Murthy and Vidyasagar, 2013. |
| Leaves | Kalaktang, Arunachal Pradesh, India | Chong | Monpa | Bone fracture | Paste | Topical | Namsa et al., 2011 |
|  | Javadhu hills, Tamilnadu, India | Vellai poondu | Local people | Diabetes | Juice | Oral | Thirumalai et al., 2012 |
|  | Riverside of Navarra, Iberian Peninsula | Ajo | Basque | Herpes | To warm with oil and direct application | Topical | Calvo et al., 2011 |
|  | Alaşehir, Turkey | Sarımsak, sarımsak | Balkan, Romans,  Yürük | Diabetes, cold, flu | Paste with bulb and flowering branch | Oral | Sargın et al., 2013 |
|  | Oyo, Ogun, Osun state, Southwestern Nigeria | Ayuu | Yoruba | Female infertility | *Aframomum melegueta* (seed, Zingiberaceae), *Piper guineense* (fruit, Piperaceae), *Xylopia aethiopica* (seed, Annonaceae), *Microdesmis puberula* (leaf, Pandaceae), *Elaeis guineensis* (fruit, Arecaceae), *Capsicum annuum* (fruit, Solanaceae) ground together and cooked with catfish, palm oil, salt | Eaten as meal | Soladoye et al., 2014 |
|  | Kosovo, Southeastern Europe | Hudhra | Albanian | Toothache | Decoction | Topical | Mustafa et al., 2012b |
| Rhizomes | Kalaktang, Arunachal Pradesh, India | Chong | Monpa | Bone fracture | Juice | Topical | Namsa et al., 2011 |
|  | Batan Island, the Philippines | Acos | Ivatan, Yami | High blood pressure | Two raw or half-cooked bulbs and made a decoction with calamansi | Oral | Abe and Ohtani, 2013 |
| Fruits | Laniba village, South Western Nigeria | Aayu | Yoruba | Hypertension | Add *Allium cepa* (Amaryllidaceae), cut into pieces, then macerated in water for about three days, extract prepared | Taken orally three times daily | Sonibare and Abegunde, 2012 |
|  | Ogun State, Nigeria | Ayu | Yoruba | Rheumatoid-arthritis | Mixed with *Alstonia boonei* bark (Apocynaceae), *Citrus aurantiifolia* (10 fruits, Rutaceae), *Citrus paradisi* fruits (Rutaceae), *Zingiber officinale* (Zingiberaceae), *Allium cepa* (Amaryllidaceae) and then made a decoction | Oral | Ogbole et al., 2010 |
|  |  |  |  | Pruritus | Added with *Celosia argentea* roots (Amaranthaceae), *Hymenostegia afzelii* (Fabaceae), *Uvaria chamae* (Annonaceae), *Agelaea pentagyna* (= *A. obliqua*, Connaraceae), *Dioclea hexandra* (= *D. reflexa*, Fabaceae), *Piper guineensis* (Piperaceae), *Aframomum melegueta* (Zingiberaceae), salt, potash and made a powder | Oral |  |
|  | Bhandara district, Maharashtra, India | Lasun | Gond | Wounds, cuts | Added with the fruit of *Semecarpus anacardium* (Anacardiaceae), the resin of *Gardenia tubifera* (= *G. resinifera*, Rubiaceae) and warmed in castor oil | Topical | Gupta et al., 2010 |
|  | Balaghat district, Madhya Pradesh, India | Lasun | Baigas, Gond, Korku | Kidney stone | Decoction with the root of *Rotheca serrata* (= *Clerodendrum serratum*, Lamiaceae) and fruit of *Datura metel* (Solanaceae) | Oral | Jain et al., 2011 |
| Root | Iran | Sir | Persi | Hypertension | Decoction | Oral | Baharvand-Ahmadi and Asadi-Samani, 2017 |
|  | Horro  Gudurru Woredas, Western Ethiopia | Qullubbii adii | Oromo | Abdominal pain | Decoction | Oral | Birhanu et al., 2015 |
|  | Batan Island, the Philippines | Acos | Ivatan, Yami | Arthritis | Decoction | Oral | Abe and Ohtani, 2013 |
|  | Ada’a district, Oromia Regional State,  Ethiopia | Qullubbi adii/ Nech shinkurt | Tulema | General malaise | Smelling roots | Topical | Kefalew et al., 2015 |
|  |  |  |  | Ascariasis | Powder with the root powder of *Ajuga integrifolia* (Lamiaceae), *Rumex nepalensis* (Polygonaceae) concocted together | Drunk once before breakfast |  |
|  |  |  |  | Flu | Root and leaf crushed into pieces, boiled with honey | Take in the liquid in a cup of tea |  |
|  |  |  |  | Toothache | Crushed, chewed, and hold between the aching tooth | Topical |  |
|  |  |  |  | Malaria | Root with leaf of *Vernonia amygdalina* (Asteraceae) pounded | Drunk the extracted solution |  |
|  | Amathole District,  Eastern Cape Province, South Africa | Ivimbampunzi | IsiXhosa | Broad-spectrum antibiotics, hypertension, gastrointestinal disorders, cough | Decoction | Oral | Otang et al., 2012 |
|  | Asgede Tsimbila district, Tigray region, Ethiopia | Tsaedashingurti | Gedeo, Amhara, Oromo, Konta, Zay | Herpes virus, cough | Decoction | Oral | Zenebe et al., 2012 |
|  | Harar, Haramaya, Bati and Garamuleta, Eastern Ethiopia | Nechshinkurt | Oromo | Abdominal  discomfort,  diarrhoea,  headache | The root is chopped and socked with hot water or tea | Taken  orally | Mekonnen and Abebe, 2017 |
| Seed | Horro  Gudurru Woredas, Western Ethiopia | Qullubbii adii | Oromo | Skin  disease | The fresh or dried root of *Kalanchoe laciniata* (Crassulaceae), the seed of *Capsicum frutescens* (Solanaceae), and the leaves of *Croton macrostachyus* (Euphorbiaceae) powdered together and mixed with water | Topical | Birhanu et al., 2015 |
|  | Jhansi district, Uttar Pradesh, India | Lashon | Sahariya | Bronchitis | Ground with the bulb of *Allium cepa* (Amaryllidaceae) | Nasal | Nigam and Sharma, 2010 |
| Stem | Cappadocica, Nevşehir, Turkey | Sarımsak | Turkish | Antiseptic, appetizing,  hypertension | Crude | Oral, topical | Akgül et al., 2016 |
|  | Tata Province, Morocco | Thouma – Tiskrt | Amazigh, Jews, Saharan, Arab-Muslim | Migraine, headache | Fumigation | Inhalation | Abouri et al., 2012 |
